# Supplementary material for: A Bayesian approach to infer recombination patterns in coronaviruses
Source: Nat Commun. 2022 Jul 20;13:4186. doi: 10.1038/s41467-022-31749-8 (PMC9297283; doi:10.1038/s41467-022-31749-8)
Supplement: Supplementary file 1 — Supplementary Information [file 41467_2022_31749_MOESM1_ESM.pdf]

Supplementary Information: A Bayesian coalescent approach to detect recombination patterns in coronaviruses

Nicola F. Müller[1,*], Kathryn E. Kistler[1,2], Trevor Bedford[1,2,3],

[1]Vaccine and Infectious Disease Division, Fred Hutchinson Cancer Research Center, Seattle, WA, USA
[2]Molecular and Cellular Biology Program, University of Washington, Seattle, WA, USA
[3]Howard Hughes Medical Institute, Seattle, WA, USA

[*]Corresponding author

Contact: nicola.felix.mueller@gmail.com

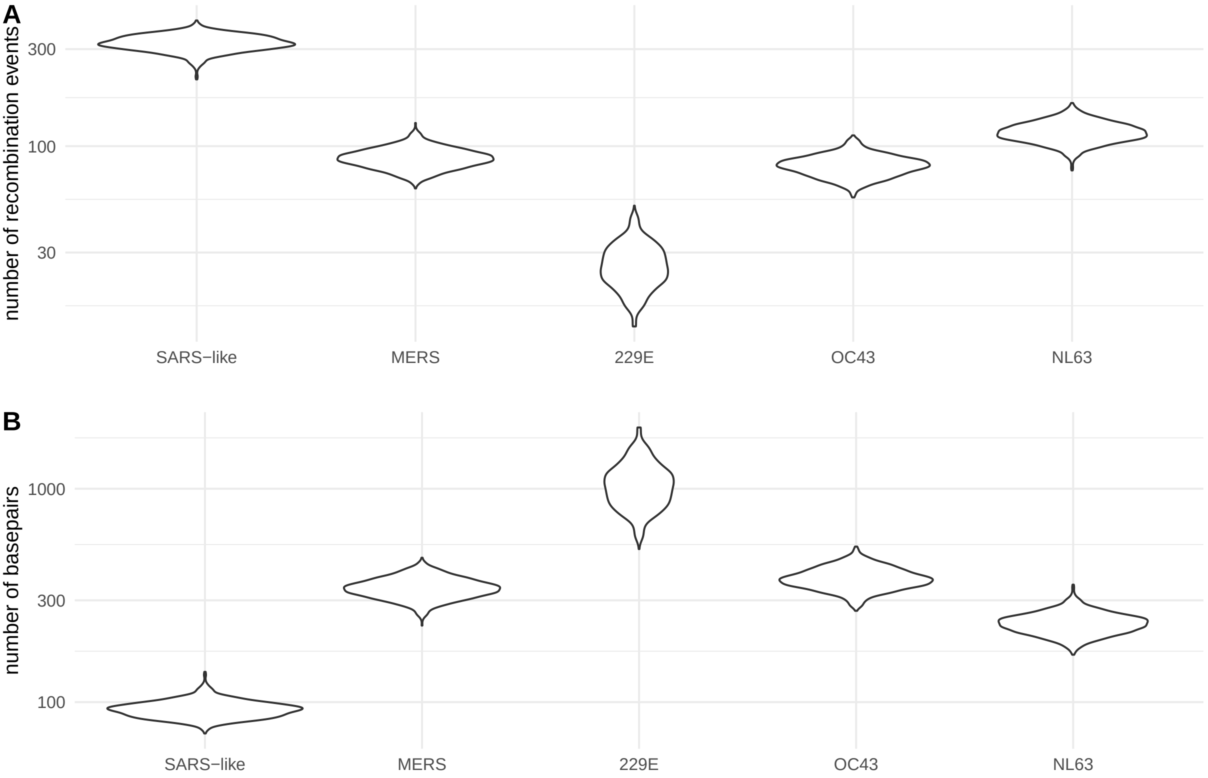

Figure S1: **Number of observable recombination events and average length of genomic segment coding for the same tree. A** Number of recombination events that impact the genome of sampled viruses in the dataset. **B** Average length of a segment in the genome of sampled viruses in the dataset that code for the same phylogenetic tree. That is the average length of a part of the genome that is not broken up by recombination events.

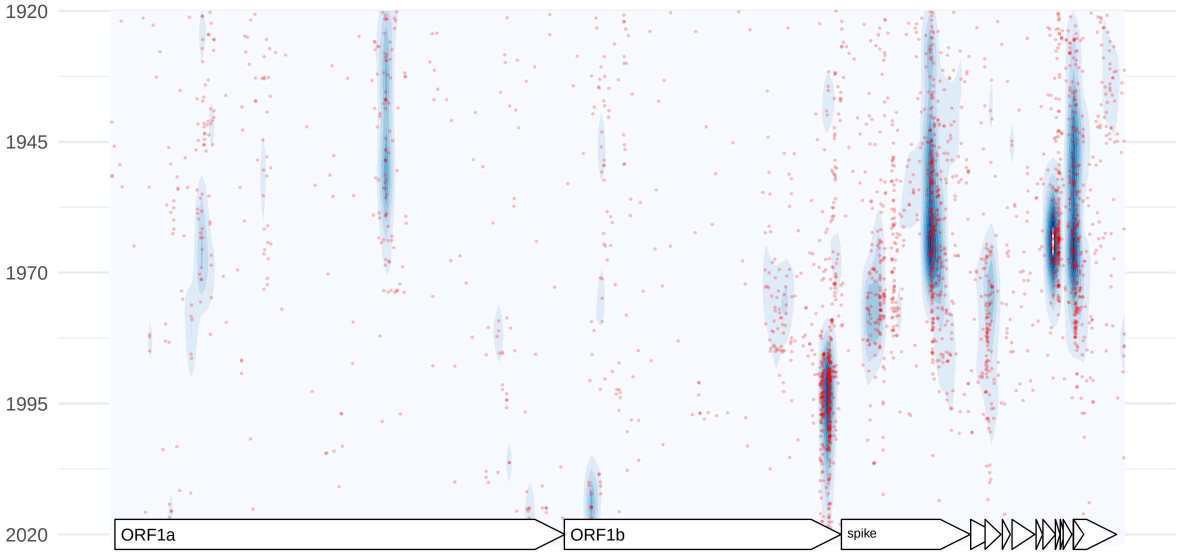

Figure S2: **Inferred timings and locations of recombination events ancestral to SARS-CoV-2 in the last one hundred years.** Timings and positions of inferred recombination events ancestral to the SARS-CoV-2 lineage are plotted. Each red dot denotes one event in the posterior distribution with the genome position on the x-axis and the year on the y-axis. Each dot represents a probability weight of 0.002% for a recombination event, meaning that 500 dots corresponds to a probability weight of 1 for an event. The density of these events is shown by a contour plot.

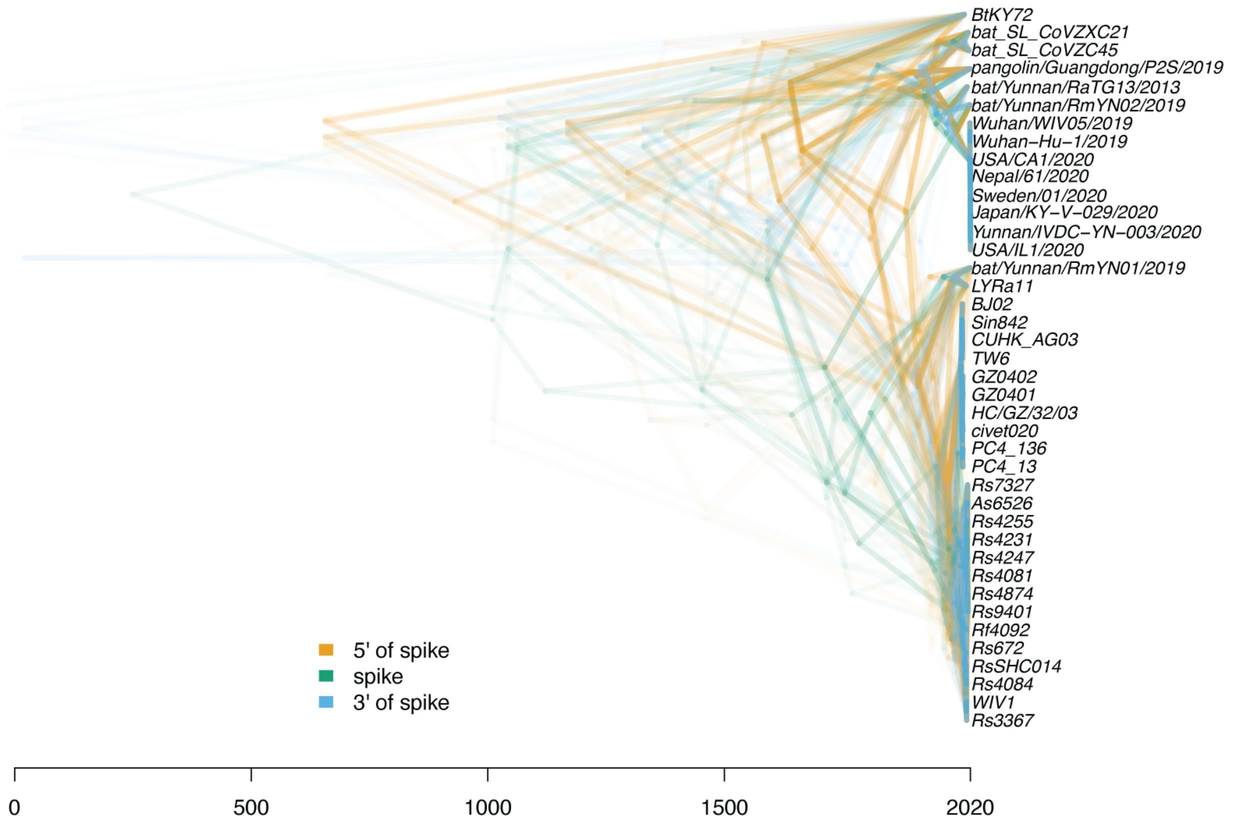

Figure S3: **Plot of the local trees of SARS-like virus on different positions across the genome.** Densitree (Bouckaert, 2010) plot of local trees in the mcc network of SARS-like viruses. The local trees are shown for every 100th position in the genome and are computed from the mcc network shown in Fig. 1A. The different colors represent whether a local trees was towards the 5' or 3' end relative to the region that codes for the spike protein, or whether it was on spike itself.

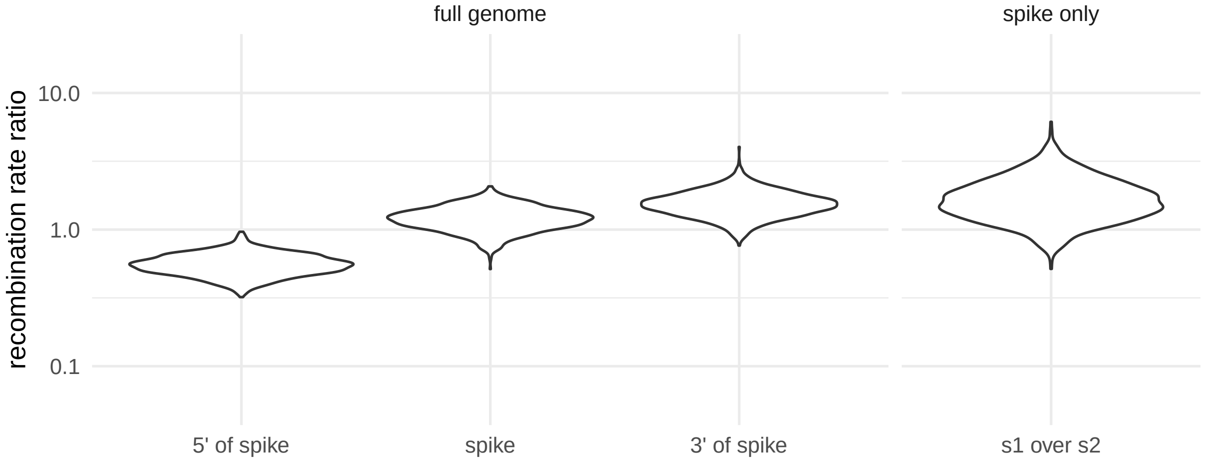

Figure S4: **Recombination rate ratios of SARS-like viruses on different parts of the genome.** Recombination rate ratios for SARS-like viruses based on two different analyses: one using the full genome (left) and one using the spike protein only (right). The rate ratios denote the rate on a part of the genome divided by the average rate on the two other parts of the genome. s1 over s2 denotes the rate ratio on spike subunit 1 over subunit 2.

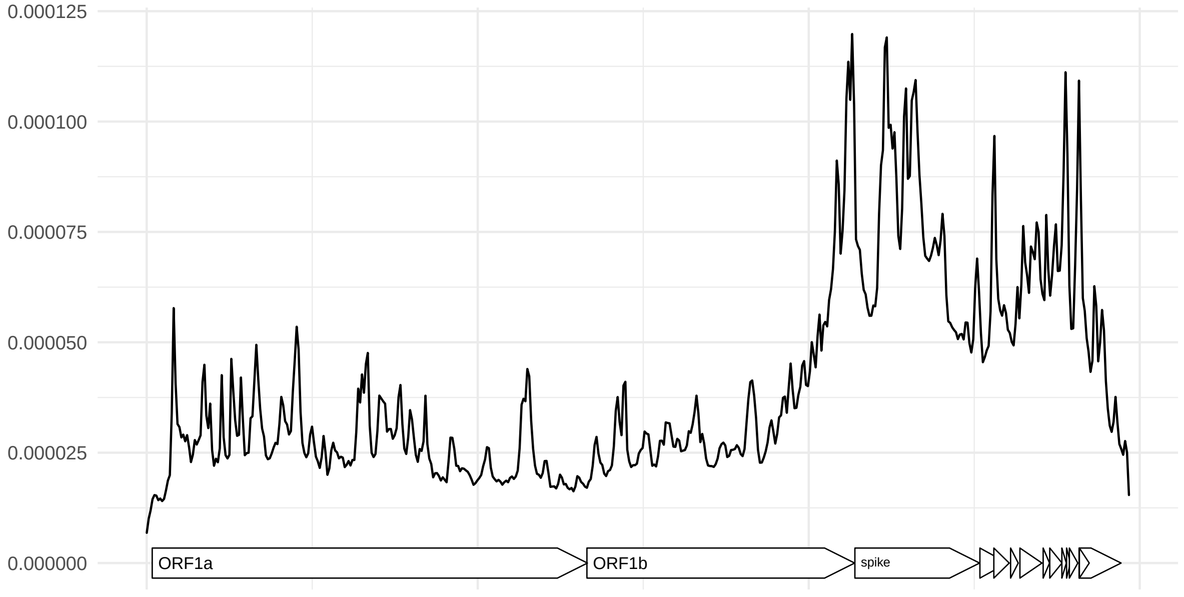

Figure S5: **Inferred locations of recombination events in the SARS-like dataset.** Here, we show the probability density of recombination events (on the y-axis) along the genome (on the x-axis) of SARS-like viruses.

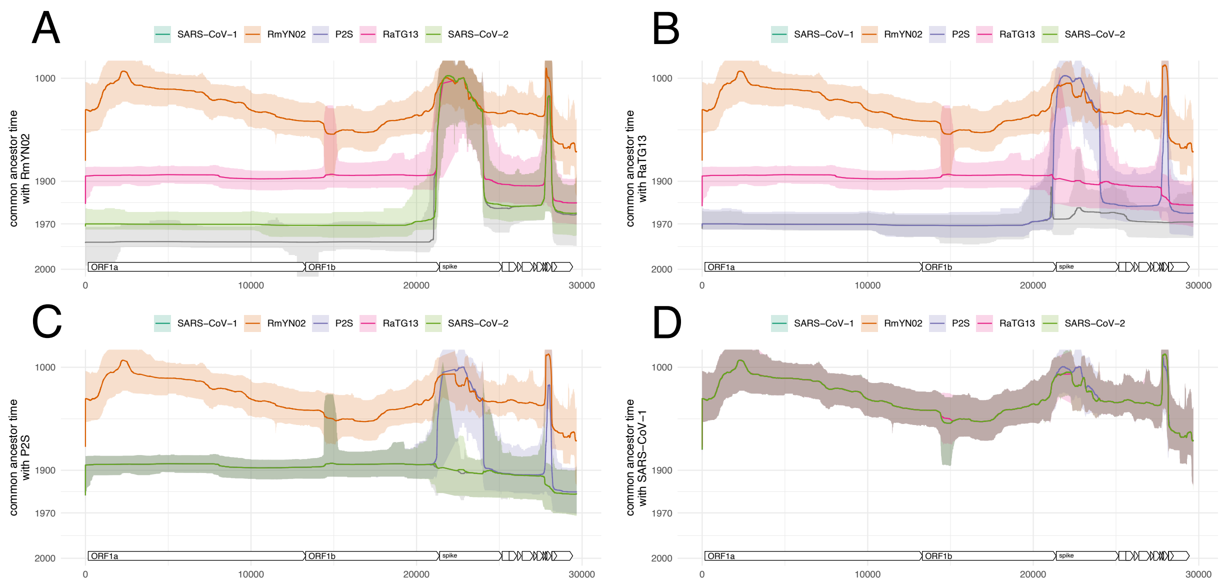

Figure S6: **Common ancestor times between sequences of the SARS-CoV-2 clade, as well as SARS-CoV-1.** Estimate of common ancestor times of RmYN02 (**A**), RaTG13 **B**, P2S **C** and SARS-CoV-1 **D** with each other and with SARS-CoV-2. The estimates of the common ancestor times assume an evolutionary rate of 5×10^{-4}. Lower rates would push the common ancestor times further into the past, while higher rates would bring the closer to the present. The line denotes the median common ancestor time, while the colored area denotes the 95% highest posterior density interval.

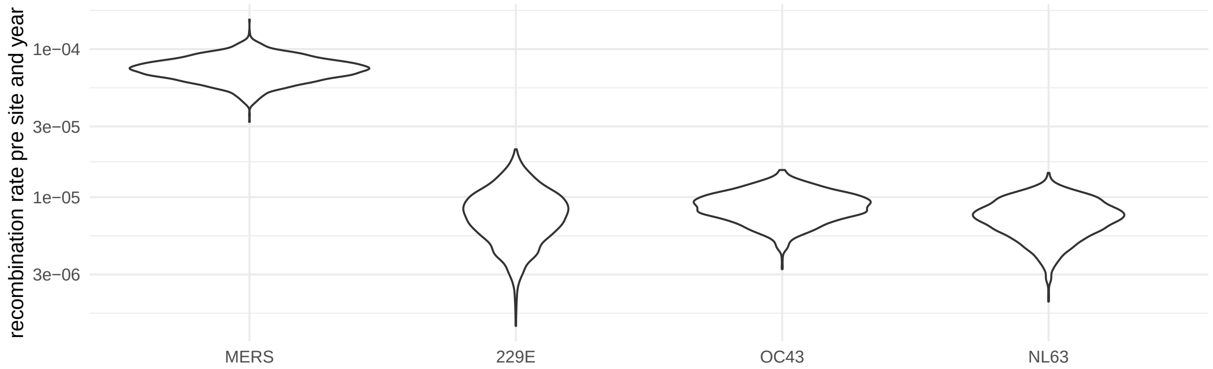

Figure S7: **Inferred recombination rates for the different coronaviruses.** Posterior distribution of recombination rates per lineage, per year and per pair of adjacent nucleotides.

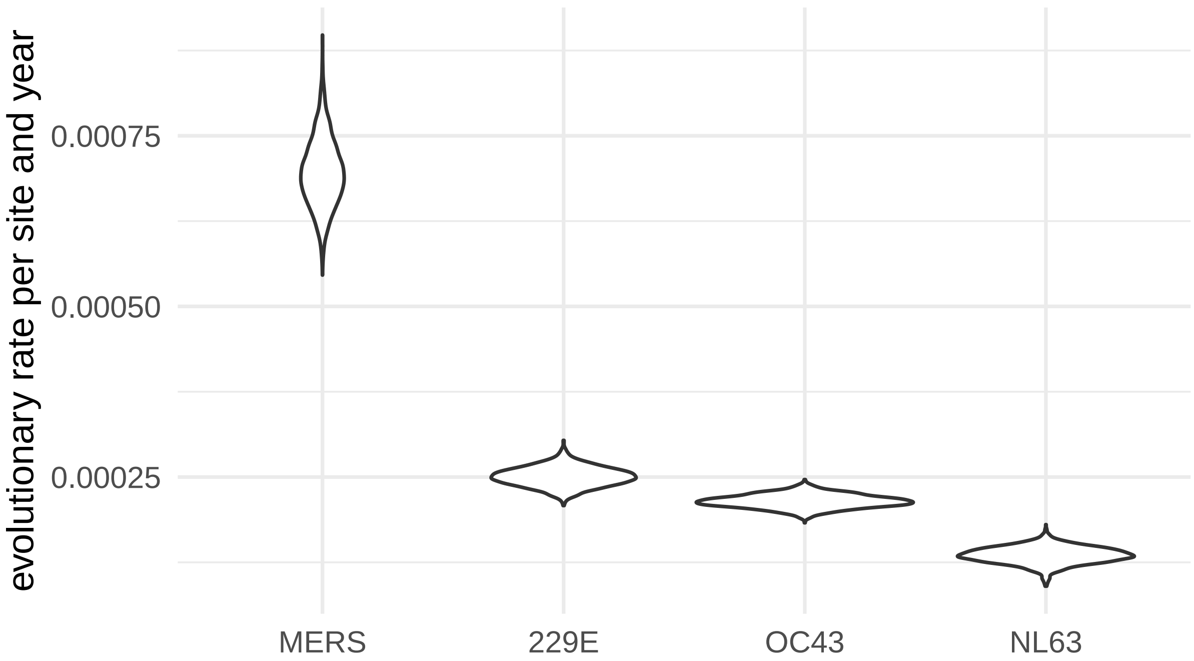

Figure S8: **Inferred evolutionary rates for the different coronaviruses.** Posterior distribution of evolutionary rates per year and nucleotide.

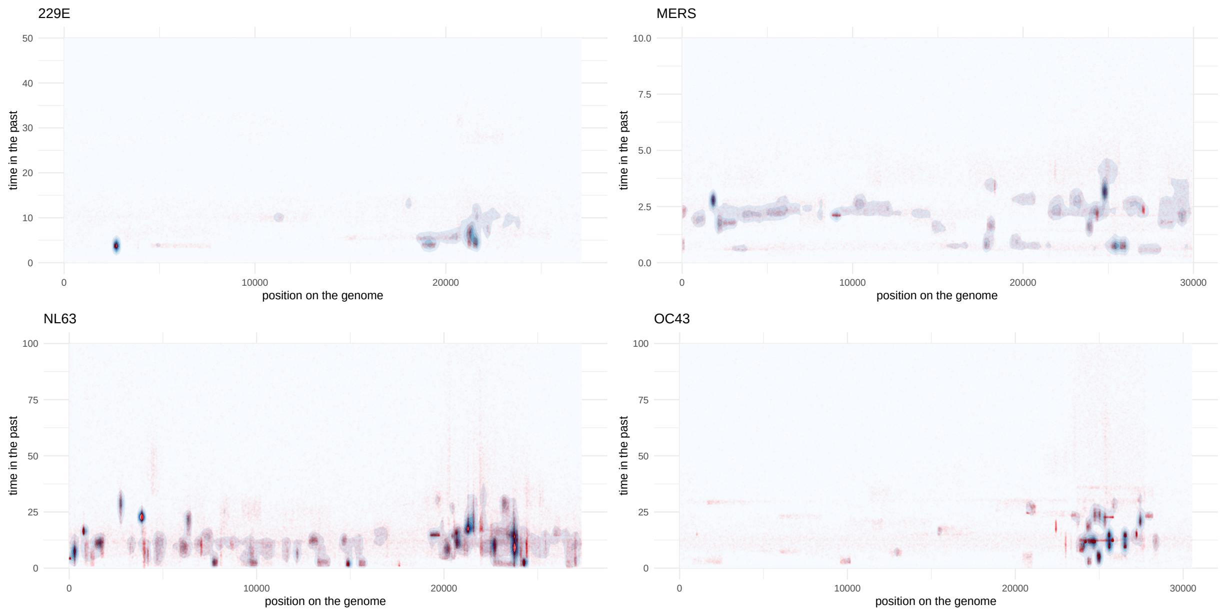

Figure S9: **Inferred timings and locations of recombination events of MERS, 229E, OC43 and NL63.** Each red dot denotes one event in the posterior distribution with the genome position on the x-axis and the year on the y-axis. The density of these events is shown by a contour plot.

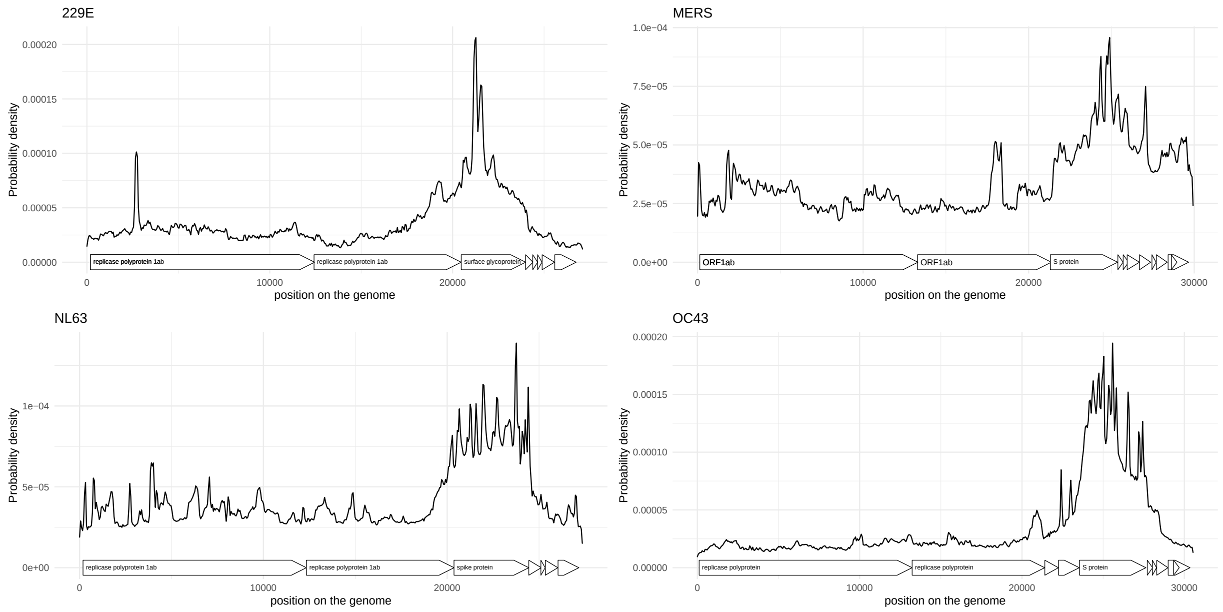

Figure S10: **Inferred locations of recombination events of MERS, 229E, OC43 and NL63.** Here, we show the probability density of recombination events (on the y-axis) along the genome (on the x-axis) for different coronaviruses.

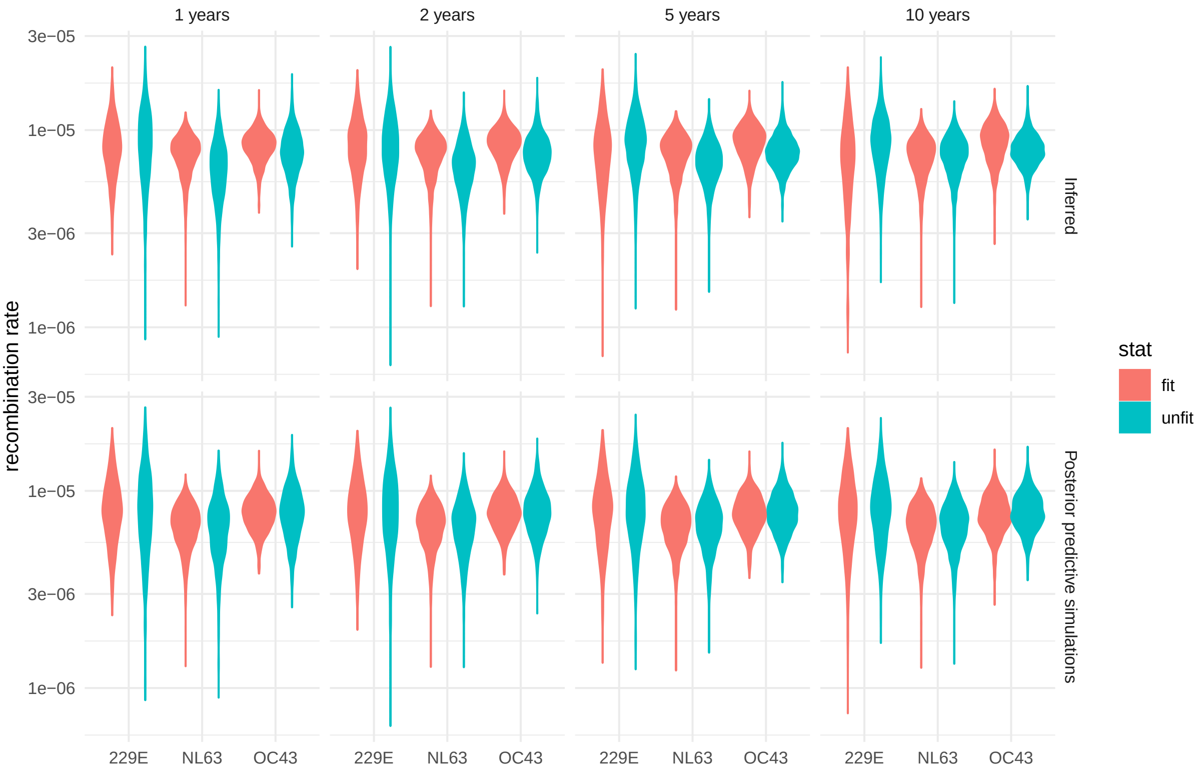

Figure S11: **Recombination rates of different parts of the recombination networks.** Recombination rates are computed for different parts of the network based on how long lineages persist for into the future. For this analysis, we classified each edge of the recombination network in the posterior distribution into fit and unfit. Fit are edges that persist for at least 1, 2, 5 or 10 years into the future (plots from left to right). We compute the rates of recombination on these edges as well as on those who go extinct more rapidly. We repeat the same for posterior predictive recombination networks that we simulated from the given sampling times, the inferred effective population sizes and the inferred recombination rates under the coalescent with recombination.

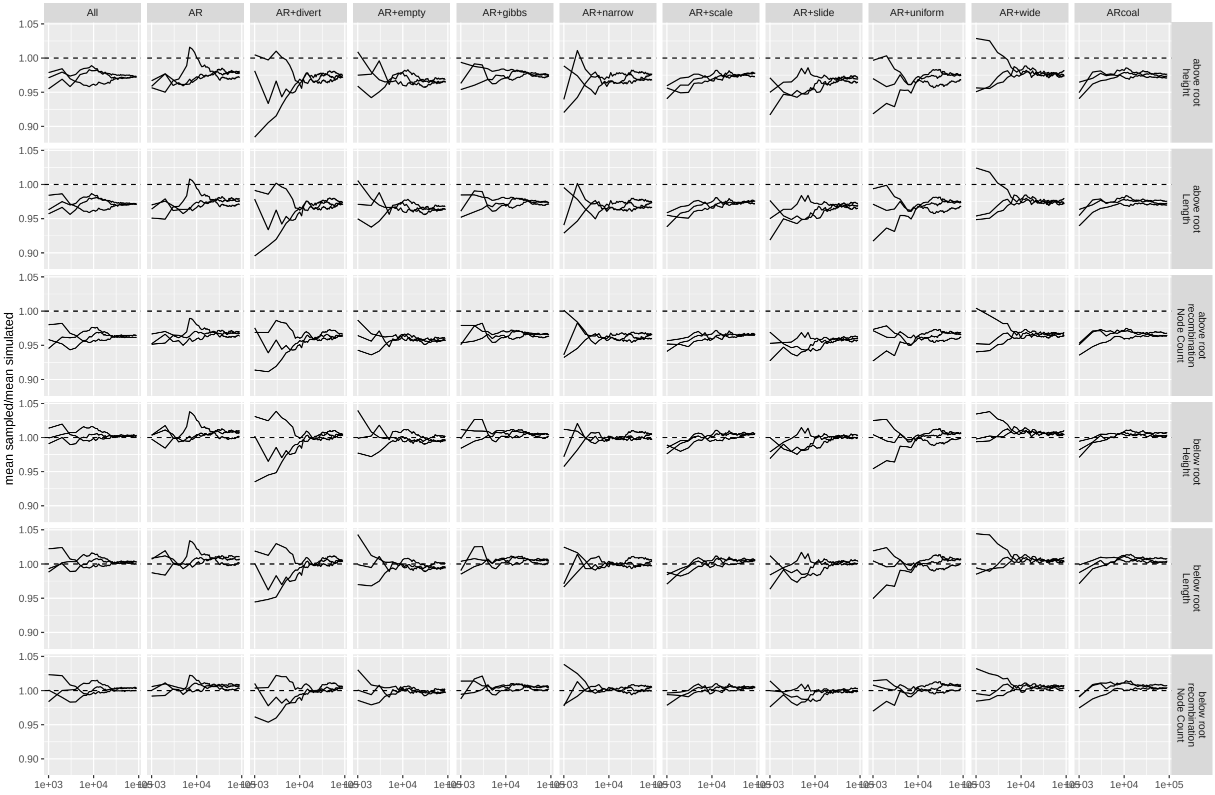

Figure S12: **Comparison of network statistics when simulating under the coalescent with recombination compared to sampling under the truncated coalescent with recombination.** We compare the posterior distributions of network height, length and the number of recombination nodes when simulating recombination networks under th coalescent with recombination and when MCMC sampling under the implementation of coalescent with recombination. We compare this for all the different MCMC operators implemented. For MCMC operators which are not universal (cannot reach every point in the posterior distribution by themselves), we tested the operator jointly with the Add/remove operator. The statistics above the root take into account the full distribution of networks. The statistics below the root only take into account the parts of the network that are below (more recent) than the oldest root of any individual position in the alignment. These are the parts of the network that directly impact the likelihood.

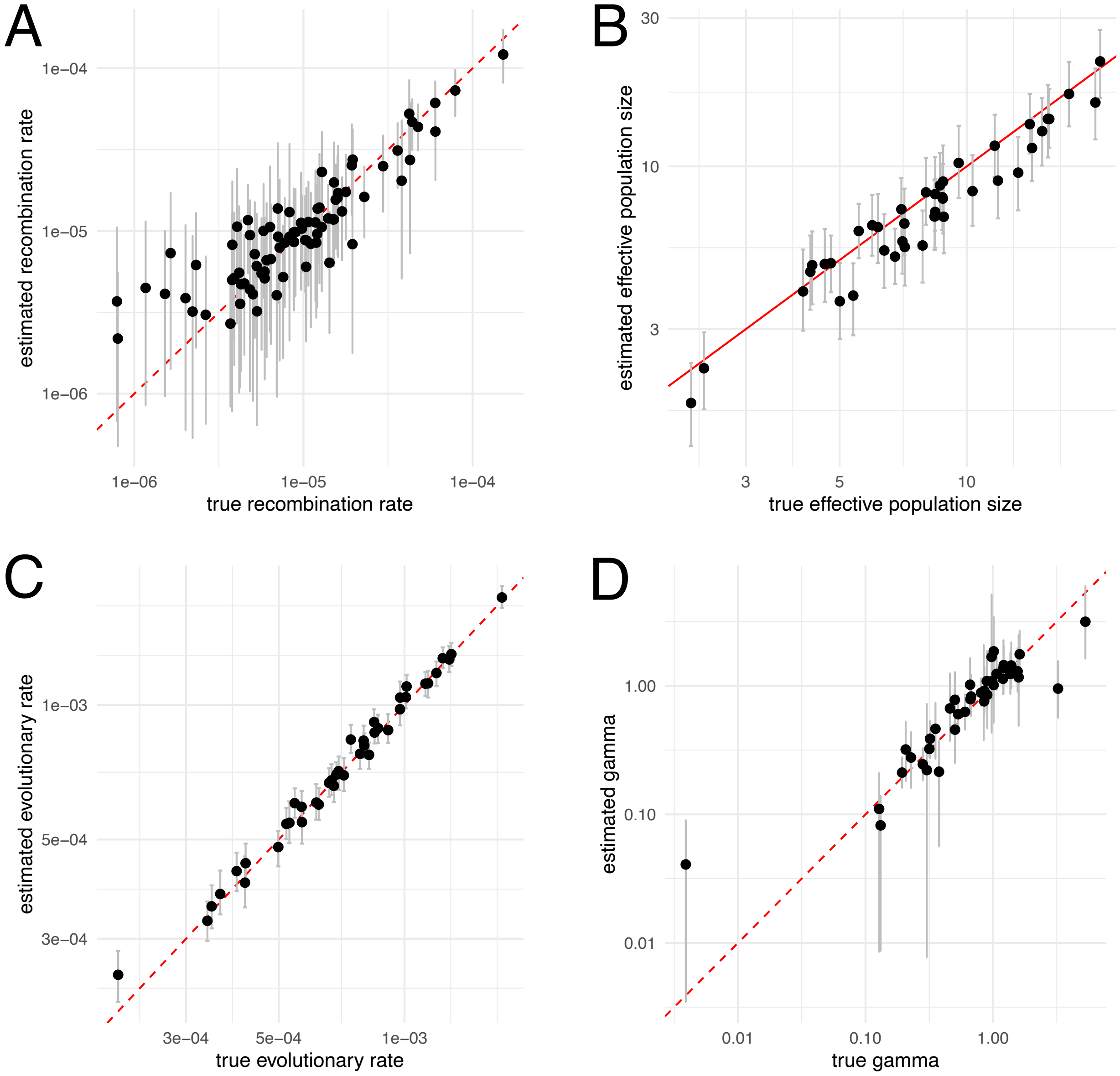

Figure S13: **Inferred vs. true rates based on simulated data. A** Simulated/true recombination rate on the x-axis compared to the estimated recombination rate on the y-axis. The points are the median estimates, while the errorbars denote the lower and upper bounds of the 95% highest posterior density intervals. **B** Simulated/true vs. estimated effective population sizes. **C** Simulated/true vs. estimated evolutionary rate. **D** Simulated/true vs. estimated gamma of the HKY+Γ_4 site model.

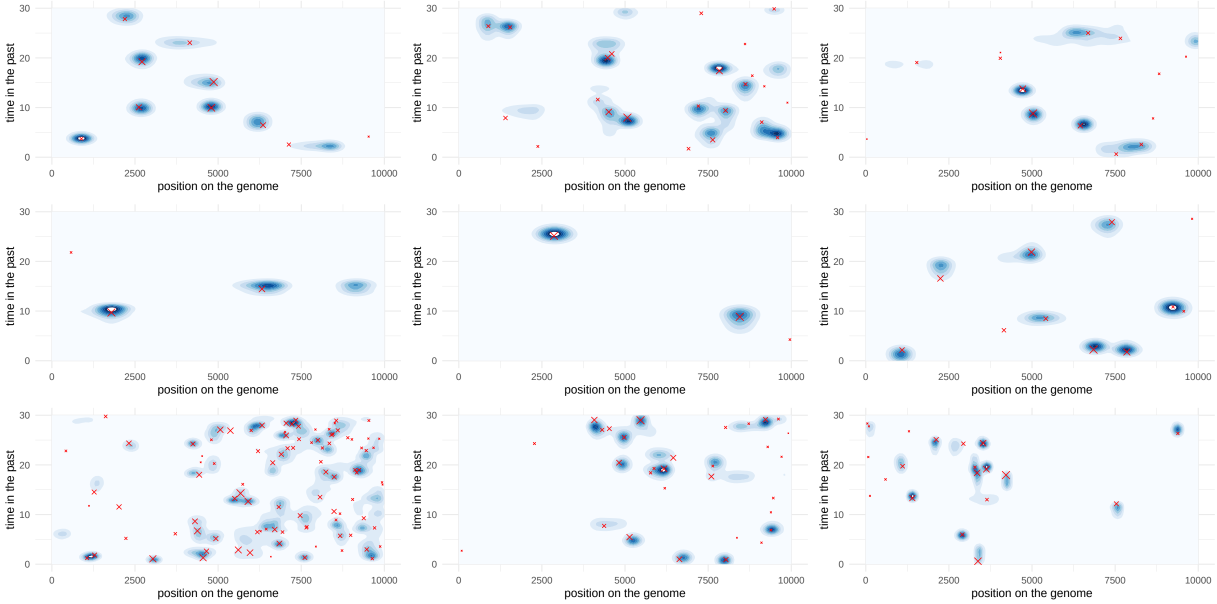

Figure S14: **Inferred vs. simulated recombination events.** Here, we show the recombination events for the simulated networks (red cross) with the position on the genome on the x-axis and the timing of the event on the y-axis. The contour plots show the density for inferred recombination events for the first 9 iterations of the simulation study. The colors are scaled such that the peak intensity has the same color in all plots. The time in the past is limited to the duration of sampling, i.e. the time when samples were taken.

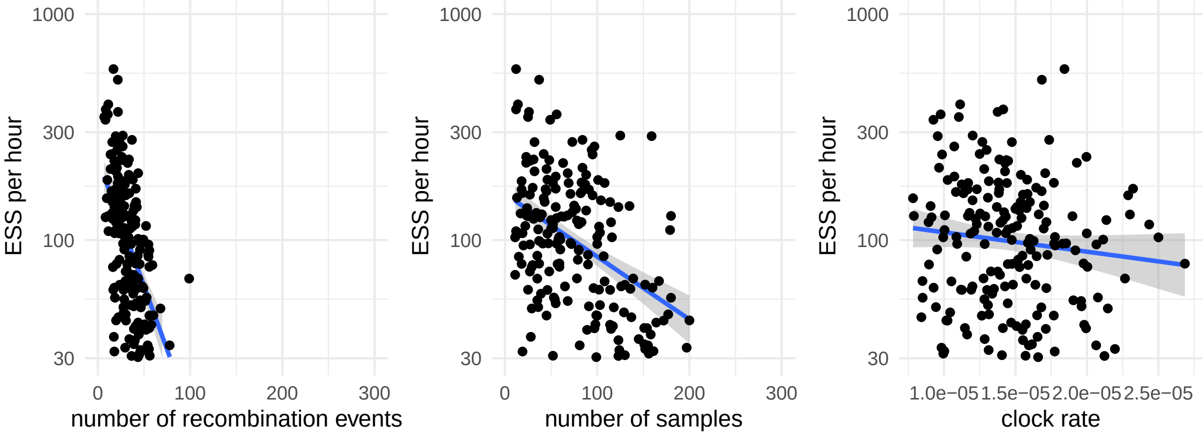

Figure S15: **Effective sample sizes per megasample.** We computed the effective sample size values computed using coda (Plummer *et al.*, 2006) for posterior probabilities, network/tree likelihood values, network/tree root heights and effective population sizes for 300 simulations. The line denotes the mean estimate of the linear regression and the shaded area the 95% confidence interval of the linear regression.

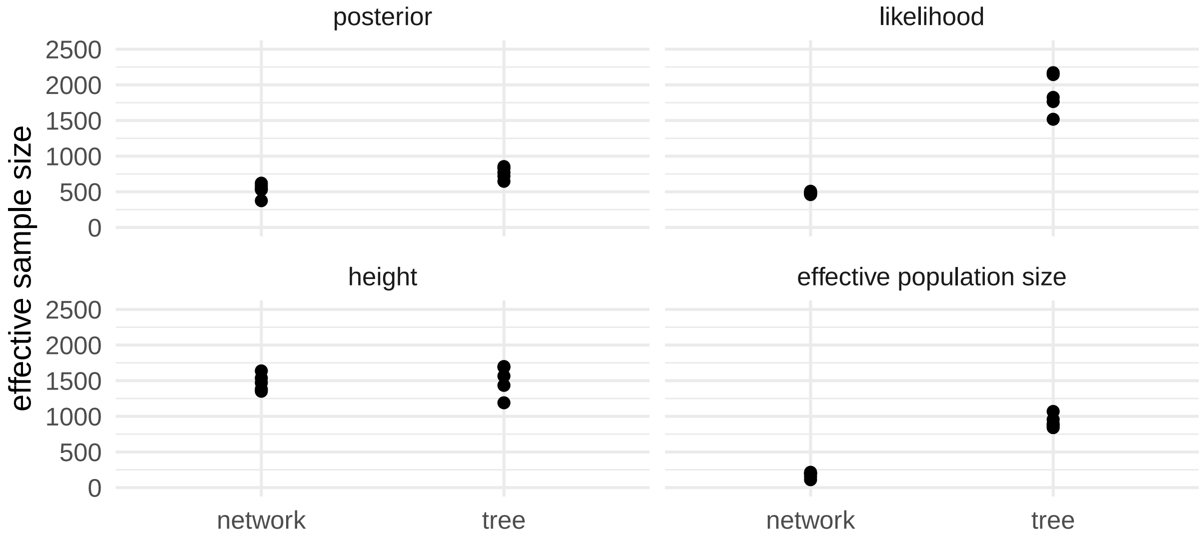

Figure S16: **Effective sample Sizes of MERS MCMC runs using the spike protein only.** Here, we compare ESS values after 25 Million MCMC iterations when inferring either networks or considering trees only for 100 MERS spike sequences. The operator weights for the inference of recombination networks is the same as used in the other coronaviruses in this manuscript. For the tree inferences, we used the default operator weights. We computed the effective sample size values computed using coda (Plummer *et al.*, 2006) for posterior probabilities, network/tree likelihood values, network/tree root heights and effective population sizes.

Supplementary Note 1

We gratefully acknowledge the following Authors from the Originating laboratories responsible for obtaining the specimens, as well as the Submitting laboratories where the genome data were generated and shared via GISAID, on which this research is based.

All Submitters of data may be contacted directly via www.gisaid.org

Authors are sorted alphabetically.

Acknowledgement EPI_SET Identifier: EPI_SET_20220531ey

Accession ID	Originating Laboratory	Submitting Laboratory	Authors
EPI_ISL_410544	Beijing Institute of Microbiology and Epidemiology	Beijing Institute of Microbiology and Epidemiology	Bao-Gui Jiang; Jia-Fu Jiang; Na Jia; Tommy Tsan-Yuk Lam; Wu-Chun Cao; Ya-Wei Zhang
EPI_ISL_412976, EPI_ISL_412977	Shandong First Medical University & Shandong Academy of Medical Sciences	Institute of Microbiology, Chinese Academy of Sciences	Alice Catherine Hughes; Hong Zhou; Juan Li; Tao Hu; Weifeng Shi; Xing Chen; Yuhai Bi
EPI_ISL_402131	Wuhan Institute of Virology, Chinese Academy of Sciences	Wuhan Institute of Virology, Chinese Academy of Sciences	Bei Li; Ben Hu; Hao-Rui Si; Peng Zhou; Ping Yu; Xing-Lou Yang; Yan Zhu; Zheng-Li Shi

References

Bouckaert, R. R. (2010). Densitree: making sense of sets of phylogenetic trees. *Bioinformatics*, **26**(10), 1372–1373.

Plummer, M., Best, N., Cowles, K., and Vines, K. (2006). Coda: convergence diagnosis and output analysis for mcmc. *R news*, **6**(1), 7–11.
