# Supplementary material for: A Bayesian approach to infer recombination patterns in coronaviruses
Source: Nat Commun. 2022 Jul 20;13:4186. doi: 10.1038/s41467-022-31749-8 (PMC9297283; doi:10.1038/s41467-022-31749-8)
Supplement: Supplementary file 3 — Description of Additional Supplementary Files [file 41467_2022_31749_MOESM3_ESM.pdf]

Description of Additional Supplementary Files

File Name: Supplementary Data 1
Description: Genbank accession numbers for the 229E, OC43, NL63, SARS-like and MERS analyses.
