# Supplementary material for: A Bayesian approach to infer recombination patterns in coronaviruses
Source: Nat Commun. 2022 Jul 20;13:4186. doi: 10.1038/s41467-022-31749-8 (PMC9297283; doi:10.1038/s41467-022-31749-8)
Supplement: Supplementary file 5 — Reporting Summary [file 41467_2022_31749_MOESM5_ESM.pdf]

nature portfolio

Corresponding author(s): Nicola F. Müller

Last updated by author(s): Jun 1, 2022

Reporting Summary

Nature Portfolio wishes to improve the reproducibility of the work that we publish. This form provides structure for consistency and transparency in reporting. For further information on Nature Portfolio policies, see our Editorial Policies and the Editorial Policy Checklist.

Statistics

For all statistical analyses, confirm that the following items are present in the figure legend, table legend, main text, or Methods section.

n/a	Confirmed	
☐	☒	The exact sample size (*n*) for each experimental group/condition, given as a discrete number and unit of measurement
☒	☐	A statement on whether measurements were taken from distinct samples or whether the same sample was measured repeatedly
☒	☐	The statistical test(s) used AND whether they are one- or two-sided<br>*Only common tests should be described solely by name; describe more complex techniques in the Methods section.*
☒	☐	A description of all covariates tested
☒	☐	A description of any assumptions or corrections, such as tests of normality and adjustment for multiple comparisons
☐	☒	A full description of the statistical parameters including central tendency (e.g. means) or other basic estimates (e.g. regression coefficient) AND variation (e.g. standard deviation) or associated estimates of uncertainty (e.g. confidence intervals)
☒	☐	For null hypothesis testing, the test statistic (e.g. *F*, *t*, *r*) with confidence intervals, effect sizes, degrees of freedom and *P* value noted<br>*Give P values as exact values whenever suitable.*
☐	☒	For Bayesian analysis, information on the choice of priors and Markov chain Monte Carlo settings
☒	☐	For hierarchical and complex designs, identification of the appropriate level for tests and full reporting of outcomes
☒	☐	Estimates of effect sizes (e.g. Cohen's *d*, Pearson's *r*), indicating how they were calculated

Our web collection on statistics for biologists contains articles on many of the points above.

Software and code

Policy information about availability of computer code

Data collection	The Recombination package is implemented as an addon to the Bayesian phylogenetics software platform BEAST2.<br>All MCMC analyses performed here, were run using adaptive parallel tempering.<br>The source code is available at https://github.com/nicfel/Recombination. We additionally provide a tutorial on how to setup and postprocessed an analysis at https://github.com/nicfel/Recombination-Tutorial.
Data analysis	The MCC networks are plotted using an adapted version of baltic (https://github.com/evogytis/baltic) that is part of the repository https://github.com/nicfel/Recombination-Material and https://doi.org/10.5281/zenodo.6600818.<br>All other plots are done in R 4.2.2 using ggplot2 3.3.6 and gggenes 0.4.0.<br>Matlab scripts were run using MATLAB_R2019b

For manuscripts utilizing custom algorithms or software that are central to the research but not yet described in published literature, software must be made available to editors and reviewers. We strongly encourage code deposition in a community repository (e.g. GitHub). See the Nature Portfolio guidelines for submitting code & software for further information.

Data

Policy information about availability of data

All manuscripts must include a data availability statement. This statement should provide the following information, where applicable:

- Accession codes, unique identifiers, or web links for publicly available datasets
- A description of any restrictions on data availability
- For clinical datasets or third party data, please ensure that the statement adheres to our policy

The BEAST2 input xml files for all coronavirus analyses in this manuscript, as well as the files used to post process these analyses are available from https://github.com/nicfel/Recombination-Material and here https://doi.org/10.5281/zenodo.6600818. The xml files include the sequence data and exact input specification of the coronavirus analyses performed in this manuscript, except for the sequences published on gisaid. The acknowledgment table for the four gisaid sequences used for the SARS-like analyses is provided in the supplement. The genbank accession numbers for the 229E, OC43, NL63, SARS-like and MERS analyses are provided as separate tables in the supplement. The MERS sequences without accession numbers are used from Dudas et al., 2018, eLife.

Field-specific reporting

Please select the one below that is the best fit for your research. If you are not sure, read the appropriate sections before making your selection.

[x] Life sciences [ ] Behavioural & social sciences [ ] Ecological, evolutionary & environmental sciences

For a reference copy of the document with all sections, see nature.com/documents/nr-reporting-summary-flat.pdf

Life sciences study design

All studies must disclose on these points even when the disclosure is negative.

Sample size	If more than 100 sequences were available for a coronavirus dataset (MERS, NL63 and OC43), 100 random sequences were used. Otherwise, the full dataset was used (54 sequences for the 229E dataset).
Data exclusions	For the MERS, NL63, OC43 and 229E dataset, sequences were excluded if they had N's on more than 25% of positions.
Replication	Publicly available sequences were used, as such, the sequencing itself can't be replicated
Randomization	Either random subsets of the available sequence data were used, or, if not enough sequence data was available, all available sequences were used
Blinding	We analysed publicly available sequence data

Reporting for specific materials, systems and methods

We require information from authors about some types of materials, experimental systems and methods used in many studies. Here, indicate whether each material, system or method listed is relevant to your study. If you are not sure if a list item applies to your research, read the appropriate section before selecting a response.

Materials & experimental systems

n/a	Involved in the study
[x]	[ ] Antibodies
[x]	[ ] Eukaryotic cell lines
[x]	[ ] Palaeontology and archaeology
[x]	[ ] Animals and other organisms
[x]	[ ] Human research participants
[x]	[ ] Clinical data
[x]	[ ] Dual use research of concern

Methods

n/a	Involved in the study
[x]	[ ] ChIP-seq
[x]	[ ] Flow cytometry
[x]	[ ] MRI-based neuroimaging
